# Researches on the Development of the Muscular Fibres of the Heart

**Published:** 1850-07

**Authors:** 


*Researches on the development of the muscular fibres of the heart, and on voluntary motion.* By Dr. Lebert.—Towards the thirty-sixth hour of incubation, the contractions of the heart of the embryo chick are distinct and regular: nevertheless, at this time the structure of the heart presents only organo-plastic globules surrounded by granular matter. The primal structure of the embryo heart is the same in all vertebrate animals; and in many lower animals, such as the compound ascidia (e. g. *Amorucium*, Milne Edwards), it constitutes the permanent condition. An undoubted difference between these and the globules of the heart's structure may be established from their earliest appearance. This obtains equally with the globules of the hearts of the embryos of mammalia and of fishes: the batrachians alone seem to form an exception, and in these there is rather resemblance than identity between the two kinds of globules.

From the third to the fourth day of incubation the structure of the heart assumes a more diffused appearance. The cellular envelopes of the organo-plastic globules in great part disappear. At this time a superficial layer of fusiform elements may be distinguished, which afterwards becomes the pericardium.

From the fourth to the fifth day there may be noticed for the first time in the midst of the globular elements, elongated cylindrical bodies arranged in reticulated groups. These, the inorganic cells or bodies, constitute the first rudiments of the muscular fibres, and whether in the heart, or in the voluntary muscles, whether in the superior vertebrata or in the batrachians, these always precede the development of the true muscular fibre, which, indeed, constitutes but a higher degree of their development. M. Lebert has not been able to observe the formation of these cells or bodies by regular lineal allocation of plastic globules. Some included globules which are noticed, must be considered accidental: they are wanting in those of the embryo mammiferi, which,- however, they surround externally. The absence of nuclei and nucleoli in these bodies does not disprove their cellular character, since the same feature is found in the globules of the vitellus of the chick, and of the dorsal cord of the embryo frog. The myogenic bodies, though generally roundish, are at first irregular in form in the superior vertebrata.

After the seventh or eighth day, the plastic globules notably diminish, and entirely disappear some days later. The muscular fibres gradually assume their longitudinally striated aspect. Their internal granules arrange themselves in groups in the direction of the length of these bodies, which correspond to the *primitive fibres* of authors. The transverse markings of the fibres are not seen until much later, and it is not until toward the expiration of embryonic life that they are constantly seen. The fibres of the voluntary muscles are formed later than those of the heart, from five to six days after the heart has fully commenced its functions. To give an idea of the distance of time that separates the development of the heart from that of the muscles of voluntary motion in mammifers, it may be stated that the former is seen in the embryo of the bat when .078 of an inch in length, whereas the latter is only met with in embryos of about half an inch in length (0.468.)

A reverse order obtains in the tadpole, in which a necessity exists for early movement in the fluid in which it floats, in order to seek its nourishment. In proportion to this necessity is the early development of the voluntary muscles in all embryos.—*London Med. Gazette from Comptes Rendes.*

